# Lacrimal Sac Malignant Melanoma in 15 Japanese Patients: Case Report and Literature Review

**DOI:** 10.1177/2324709619888052

**Published:** 2019-11-20

**Authors:** Toshihiko Matsuo, Takehiro Tanaka, Osamu Yamasaki

**Affiliations:** 1 Okayama University Graduate School of Interdisciplinary Science and Engineeing in Health Systems, Okayama City, Japan; 2 Department of Ophthalmology, Okayama University Hospital, Okayama City, Japan; 3 Department of Pathology, Okayama University Graduate School of Medicine, Dentistry, and Pharmaceutical Sciences, Okayama City, Japan; 4 Department of Dermatology, Okayama University Graduate School of Medicine, Dentistry, and Pharmaceutical Sciences, Okayama City, Japan; 5 Melanoma Center, Okayama University Hospital, Okayama City, Japan

**Keywords:** lacrimal sac, malignant melanoma, BRAF inhibitor, dabrafenib, MEK inhibitor, trametinib, BRAF mutation, PET/CT

## Abstract

*Background*. Primary malignant melanoma of the lacrimal sac is rare. A patient with lacrimal sac melanoma was presented, and 14 Japanese patients with lacrimal sac melanoma in the literature were reviewed. *Case Presentation*. A 78-year-old Japanese man was presented with painless swelling of the lacrimal sac on the left side. Dacryocystectomy revealed diffuse infiltration with large epithelioid cells, sometimes with pigments, which were positive for cocktail mix of antibodies to tyrosinase, melan A (MART-1), and HMB45, leading to pathological diagnosis of melanoma. One month later, positron emission tomography (PET) revealed 2 high-uptake sites (SUV_max_ = 10.29 and 15.38) at the levels of medial canthus and nasolacrimal duct, but no abnormal uptake in the other site of the body. The lesion had the BRAF V600E mutation. He began to take daily oral dabrafenib (BRAF inhibitor) and trametinib (MEK inhibitor), leading to no abnormal uptake on PET in half a year. He had stable disease in good physical status with small and weak uptake sites of lymph nodes on PET 1 year later. *Results*. In the review of 15 Japanese patients, including this patient, local recurrence was noted in 4 patients, regional lymph node metastasis only in 3, distant metastasis in 6, and no metastasis in 6. Five patients died within 2 years and the others were alive in short follow-up periods. *Conclusions*. Chemotherapy was the standard for local recurrence or metastasis. Emerging molecular target drugs, as shown in the present patient, would change the strategy for management of lacrimal sac melanoma.

## Background

The lacrimal sac is a component of the ocular adnexa in the orbit and serves as a drainage system of the lacrimal fluid from the ocular surface through the nasolacrimal duct to the nasal cavity. The benign and malignant neoplasms arising in the lacrimal sac have been reported collectively as orbital tumors. Epithelium-derived neoplasms and lymphomas are dominant among basically rare tumors in the lacrimal sac.^1^ Malignant melanoma arising in the lacrimal sac is extremely rare, and a limited number of case reports in the literature can be listed on hand.^2-24^

In this study, we present a Japanese patient with lacrimal sac melanoma who underwent a modern combination therapy of molecular target drugs, dabrafenib (BRAF inhibitor) and trametinib (MEK inhibitor). We also reviewed 15 Japanese patients with lacrimal sac melanoma, including the present patient, who were described in the literature.^25-37^

## Case Report

**Figure 1.**
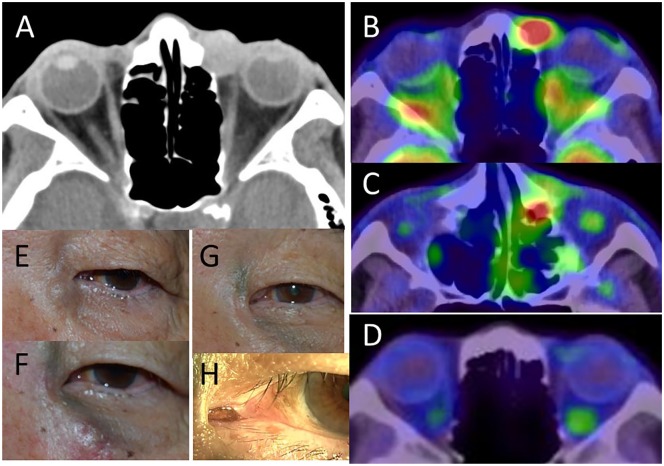
Lacrimal sac mass (E) on the left side on computed tomographic scan (A) at the initial presentation in a 78-year-old man. About 1 month after dacryocystectomy, subcutaneous pigmented lesions (F) were noted and whole-body 2-[^18^F]fluoro-2-deoxy-D-glucose positron emission tomography fused with computed tomography (PET/CT) showed 2 high-uptake sites at the levels of medial canthus (SUV_max_ = 10.29, B) and nasolacrimal duct (SUV_max_ = 15.38, C). About half a year after oral dabrafenib (Tafinlar) 300 mg daily plus trametinib (Mekinist) 2 mg daily, the subcutaneous lesions subsided (G) with unchanged conjunctival pigmentation on the lacrimal caruncle (H) and no abnormal uptake on PET/CT (D).

A 78-year-old man was presented with 10-month history of waxing and waning painless swelling of the lacrimal sac on the left side (Figure 1E). The pigmentation was noted on the lacrimal caruncle on the left side (Figure 1H). The best-corrected visual acuity was 1.0 in the right eye and 0.9 in the left eye. The ocular media and fundi in both eyes were normal. He had undergone total gastrectomy for stomach cancer 8 years previously and endoscopic colon polypectomy 4 months previously. His medication was daily amlodipine 5 mg only. Computed tomographic scan showed a 1-cm-sized round homogeneous mass with no bony change on the left lacrimal fossa (Figure 1A). He underwent left lacrimal sac tumor extirpation under general anesthesia. The lacrimal sac filled with black mass was carefully separated from the surrounding tissue, and pigmented epithelial lesions along the nasolacrimal duct were removed as deep as possible into the duct.

**Figure 2.**
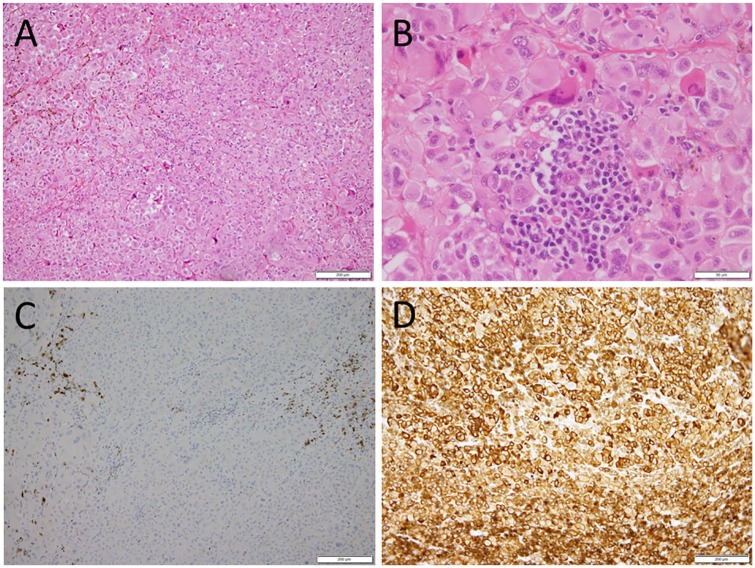
Pathology of lacrimal sac melanoma. (A) Large epithelioid cells with abnormal nuclei arranged in irregular strands and foci. (B) Small lymphocytes infiltrated as a focus among large neoplastic cells. The neoplastic cells are negative for keratin AE1/AE3 (C), and positive for cocktail-mix antibodies against tyrosinase, melan A (MART-1, melanoma antigen recognized by T cells-1), and HMB45 (D). Scale bar = 200 µm in A, C, and D. Scale bar = 50 µm in B.

The pathological examination showed malignant melanoma. The entire lacrimal sac was infiltrated with large epithelioid cells with anomalous nuclei, sometimes, multinucleated large cells in a diffuse pattern (Figure 2A and B). Pigmented abnormal cells were also present in foci. Infiltrating cells were positive for cocktail-mix antibodies against tyrosinase, melan A (MART-1, melanoma antigen recognized by T cells-1), and HMB45 (Figure 2D), but negative for cytokeratin AE1/AE3 (Figure 2C) or CAM5.2. A cluster of small lymphocytes were noted among the large neoplastic cells (Figure 2B).

One month later, he developed subcutaneous pigmented bumpy lesions surrounding the medial canthus (Figure 1F), and whole-body 2-[^18^F]fluoro-2-deoxy-D-glucose positron emission tomography fused with computed tomography (PET/CT) showed 2 high-uptake sites (SUV_max_ = 10.29 and 15.38) at the levels of the medial canthus (Figure 1B) and nasolacrimal duct (Figure 1C), with no abnormal uptake found in the other sites of the body. The lesion had the BRAF V600E mutation by a US Food and Drug Administration–approved test (the cobas 4800 BRAF V600 Mutation Test; Roche Molecular Diagnostics, Pleasanton, CA), and he showed no rise of serum 5-S-cysteinyldopa at 5.7 nmol/L (normal range = 1.5-8.0). Two months after the surgery, he began to take oral dabrafenib (Tafinlar) 300 mg daily plus trametinib (Mekinist) 2 mg daily. In half a year, the subcutaneous and nasolacrimal ductal recurrent lesions resolved (Figure 1G), and PET/CT showed no abnormal uptake in the orbit on the left side (Figure 1D), with no abnormal uptake at other sites of the body. Conjunctival pigmentation in the lacrimal caruncle on the left side (Figure 1H) remained unchanged in comparison with the initial visit. At the last visit, 1½ years after the initial visit, PET/CT showed small weak uptake sites in cervical and axillary lymph nodes. He had the stable disease in good physical status with the continuing medications at the same doses. No adverse event, except for occasional mild nausea, was noted throughout the course.

## Methods

To analyze historical cases from the literature, the Japanese literature was searched for the key words “lacrimal sac melanoma (in Japanese)” in Igaku Chuo Zasshi (Japana Centra Revuo Medicina). Old literatures were further collected from references cited in the articles identified during the literature search. PubMed was also searched for the key words “lacrimal sac melanoma.”

## Results

The 15 Japanese patients with lacrimal sac malignant melanoma, including the present patient, were 7 men and 8 women, with the age at the initial presentation ranging from 41 to 80 (median = 60) years (Table 1). The tumor was present on the right side in 6 patients, and on the left side in 8 patients, with unknown laterality in 1 patient (Case 9). Local recurrence was noted in 4 patients including the present case, regional lymph node metastasis only in 3 patients, distant metastasis in 6 patients, and no metastasis in 6 patients. As an initial surgery, all 15 patients underwent dacryocystectomy, except for 1 patient with orbital exenteration (Case 5). Lymph node dissection was done in 3 patients. Chemotherapy as a standard regimen at that time, including dacarbazine alone or combination of dacarbazine and nimustine, or further combined with vincristine or carboplatin was done in 7 patients. Additional surgeries for local recurrence were done in 2 patients. Local radiotherapy was applied to metastatic lesions, including the brain, in 2 patients. Two patients had both local recurrence and metastasis. Five patients died within 2 years and the others were alive although the follow-up periods were short. Pathologically, all patients showed epithelioid-type melanoma cells, except for one (Case 8) with spindle cell melanoma.

**Table 1.** Review of 15 Japanese Patients With Lacrimal Sac Malignant Melanoma Including the Present Patient.

Case No./Age/Gender/Laterality	Initial Surgery	Additional Treatment	Local Recurrence	Metastasis	Outcome (Follow-up After Surgery)	Author (Year)
1/50/male/right	Dacyrocystectomy	LN dissection	No	Submandibular LN; Preauricular LN	Alive (not described)	Katayama and Terada^25^ (1956)
2/41/female/left	Dacyrocystectomy	BCG immunotherapy	No	No	Alive (14 months)	Yamade and Kitagawa^26^ (1978)
3/80/female/right	Dacyrocystectomy	Additional extirpation	Yes	No	Alive (not described)	Kuwana et al^27^ (1979)
4/59/female/left	Dacyrocystectomy	Dacarbazine/nimustine; LN dissection	No	Cervical LN	Alive (5 months)	Uchida et al^28^ (1990)
5/52/female/right	Orbital exenteration	Dacarbazine/nimustine/vincristine	No	No	Alive (not described)	Takahashi et al^29^ (1991)
6/57/female/left	Dacyrocystectomy	Dacarbazine; LN dissection	No	Submandibular LN	Alive (8 months)	Matsune et al^30^ (1992)
7/78/female/left	Dacyrocystectomy	No	No	No	Alive (5 mo)	Matsuo et al^31^ (1993)
8/49/male/right	Dacyrocystectomy; Nasolacrimal duct extirpation	Chemotherapy^a^; vertebrae radiation	No	Thoracic vertebrae	Not described	Kuwabara and Takeda^32^ (1997)
9/49/male/not described	Dacyrocystectomy	Dacarbazine/nimustine/vincristine	Yes	Yes	Dead (2 years)	Goto et al^33^ (1999)
10/71/female/right	Dacyrocystectomy	Additional extirpation	Yes	Liver, rib	Dead (1 year)	Ito et al^34^ (2004)
11/60/male/right	Dacyrocystectomy	Local radiation; whole brain radiation; dacarbazine/nimustine/carboplatin	No	Preauricular LN brain, liver, spleen, mediastinum	Dead (10 months)	Shinozaki et al^35^ (2005)
12/69/male/left	Dacyrocystectomy	Local interferon-β injection	No	No	Alive (8 months)	Nakamura et al^36^ (2007)
13/80/female/left	Dacyrocystectomy	No	No	Liver, lung	Dead (not described)	Nakamura et al^36^ (2007)
14/61/male/left	Dacyrocystectomy	Dacarbazine/nimustine/vincristine; interferon-β	No	Liver, stomach, lung, brain	Dead (10 months)	Maegawa et al^37^ (2014)
15/78/male/left	Dacyrocystectomy	Dabrafenib/trametinib	Yes	No	Alive (1.5 years)	Matsuo (this case)

Abbreviations: LN, lymph node; BCG, Bacillus Calmette-Guérin.

a Not specified.

## Discussion

The present patient showed local recurrence of malignant melanoma along the nasolacrimal duct in addition to the subcutaneous infiltration. The recurrent melanoma was unresectable, and thus, a combined therapy with oral dabrafenib and trametinib was applicable since the tumor had the BRAF mutation.^38,39^ The patient maintained the stable disease with no local recurrence but with small lymphadenopathy later in one and a half year after the initial surgery of dacryocystectomy. He was in good quality of life and had only tolerable adverse event as mild nausea. In the development of distant lymphadenopathy with mild abnormal uptake on PET/CT, we decided to continue dabrafenib and trametinib and not to switch to PD-1 or PD-L1anitbodies because PD-1 or PD-L1 antibodies might cause adverse event, resulting in poor quality of life. It should be noted that foci of infiltration with small lymphocytes were present among the large melanoma cells. This fact suggests that PD-1 or PD-L1 antibodies might work in recovering the immune surveillance to neoplastic cells.

In literature review of Japanese patients with lacrimal sac melanoma, only 14 patients were found as case reports. The main features of lacrimal sac melanoma in 15 patients, including the present patient, were no dominance in gender and laterality. One third of patients had neither local recurrence nor distant metastasis after the initial surgery of dacryocystectomy. The patients with lymphadenopathy or distant metastasis or their combination usually underwent systemic chemotherapy based on dacarbazine and nimustine at that time, but died within 2 years after the initial surgery. Therefore, the prognosis was basically poor when metastasis was present.

In the emerging trend with new therapeutic drugs, BRAF inhibitor and MEK inhibitor,^38,39^ the strategy for management of lacrimal sac melanoma would be drastically changing. As complete as possible extirpation of the lacrimal sac remains the basis for local control and pathological diagnosis. In the case of nasolacrimal duct epithelial infiltration noted during the surgery, removal of pigmented lesions as deep in the nasolacrimal duct as possible would be recommended. In case of local recurrence mainly from the nasolacrimal duct lesion, further radical surgery would not be recommended and molecular target drugs would be better tried in the situation of unresectable tumor after the BRAF mutation is confirmed. Lymph node metastasis and distant metastasis are, of course, the indication of chemotherapy with molecular target drugs. Clinical staging by PET/CT is the standard in the assessment of lacrimal sac melanoma as are melanomas which arise in other sites of the body, including conjunctival melanoma^39^ and choroidal melanoma^40^ in the field of ophthalmology.

## References

[1] Krishna Y Coupland SE. Lacrimal sac tumors—a review. Asia Pac J Ophthalmol (Phila). 2017;6:173-178. 28399337 10.22608/APO.201713

[2] Duguid IM. Malignant melanoma of the lacrimal sac. Br J Ophthalmol. 1964;48:394-398. 14190454 10.1136/bjo.48.7.394 PMC505977

[3] Farkas TG Lamberson RE. Malignant melanoma of the lacrimal sac. Am J Ophthalmol. 1968;66:45-48. 5659310 10.1016/0002-9394(68)91784-4

[4] Faulborn J Witschel H. Malignant melanoma of the lacrimal sac [in German]. Klin Monbl Augenheilkd. 1972;161:662-665. 4655894

[5] Schreinzer W Breitfellner G. Primary malignant melanoma of the lacrimal sac [in German]. Klin Monbl Augenheilkd. 1980;176:262-265. 7420976 10.1055/s-2008-1057441

[6] Lloyd WC 3rd Leone CR Jr. Malignant melanoma of the lacrimal sac. Arch Ophthalmol. 1984;102:104-107. 6703953 10.1001/archopht.1984.01040030088043

[7] Glaros D Karesh JW Rodrigues MM Hirsch DR Zimmerman LE. Primary malignant melanoma of the lacrimal sac. Arch Ophthalmol. 1989;107:1244-1245. 2757556 10.1001/archopht.1989.01070020310045

[8] Eide N Refsum SB Bakke S. Primary malignant melanoma of the lacrimal sac. Acta Ophthalmol (Copenh). 1993;71:273-276. 8333278 10.1111/j.1755-3768.1993.tb05003.x

[9] Owens RM Wax MK Kostik D Linberg JV Hogg J. Malignant melanoma of the lacrimal sac. Otolaryngol Head Neck Surg. 1995;113:634-640. 7478659 10.1177/019459989511300520

[10] Levine MR Dinar Y Davies R. Malignant melanoma of the lacrimal sac. Ophthalmic Surg Lasers. 1996;27:318-320. 8705748

[11] Malik TY Sanders R Young JD Brennand E Evans AT. Malignant melanoma of the lacrimal sac. Eye (Lond). 1997;11(pt 6):935-937. 9537158 10.1038/eye.1997.232

[12] McNab AA McKelvie P. Malignant melanoma of the lacrimal sac complicating primary acquired melanosis of the conjunctiva. Ophthalmic Surg Lasers. 1997;28:501-504. 9189954

[13] Fishman G Ophir D. Malignant melanoma of the lacrimal sac: a case study. Am J Otolaryngol. 1999;20:336-339. 10512146 10.1016/s0196-0709(99)90038-8

[14] Lee HM Kang HJ Choi G, et al Two cases of primary malignant melanoma of the lacrimal sac. Head Neck. 2001;23:809-813. 11505494 10.1002/hed.1116

[15] Billing K Malhotra R Selva D Saloniklis S Taylor J Krishnan S. Magnetic resonance imaging findings in malignant melanoma of the lacrimal sac. Br J Ophthalmol. 2003;87:1187-1188. 12928297 10.1136/bjo.87.9.1187 PMC1771826

[16] Tello JS Campillo NG Rodriguez-Peralto JL Alvarez-Linera J Cocina IG. Malignant melanoma of the lacrimal sac. Otolaryngol Head Neck Surg. 2004;131:334-336. 15365559 10.1016/j.otohns.2003.09.023

[17] Gleizal A Kodjikian L Lebreton F Beziat JL. Early CT-scan for chronic lacrimal duct symptoms—case report of a malignant melanoma of the lacrimal sac and review of the literature. J Craniomaxillofac Surg. 2005;33:201-204. 15878522 10.1016/j.jcms.2005.01.012

[18] Nam JH Kim SM Choi JH, et al Primary malignant melanoma of the lacrimal sac: a case report. Korean J Intern Med. 2006;21:248-251. 17249508 10.3904/kjim.2006.21.4.248 PMC3891031

[19] Sitole S Zender CA Ahmad AZ Hammadeh R Petruzzelli GJ. Lacrimal sac melanoma. Ophthalmic Plast Reconstr Surg. 2007;23:417-419. 17882000 10.1097/IOP.0b013e31814db537

[20] Heindl LM Schick B Kampgen E Kruse FE Holbach LM. Malignant melanoma of the lacrimal sac [in German]. Ophthalmologe. 2008;105:1146-1149. 18438631 10.1007/s00347-008-1740-0

[21] Li YJ Zhu SJ Yan H Han J Wang D Xu S. Primary malignant melanoma of the lacrimal sac. BMJ Case Rep. 2012;2012:bcr2012006349. 10.1136/bcr-2012-006349 PMC4543342 22891020

[22] Pujari A Ali MJ Mulay K Naik MN Honavar SG. The black lacrimal sac: a clinicopathological correlation of a malignant melanoma with anterior lacrimal crest infiltration. Int Ophthalmol. 2014;34:111-115. 23456510 10.1007/s10792-013-9743-5

[23] Ren M Zeng JH Luo QL Bi F Chen J. Primary malignant melanoma of lacrimal sac. Int J Ophthalmol. 2014;7:1069-1070. 25540768 10.3980/j.issn.2222-3959.2014.06.30 PMC4270979

[24] Subramaniam SS Anand R Mellor TK Brennan PA. Primary lacrimal sac melanoma with metastatic cervical disease: a review of the literature and case report. J Oral Maxillofac Surg. 2017;75:1438-1441. 28215853 10.1016/j.joms.2017.01.015

[25] Katayama H Terada H. A case of the malignant melanoma occurred from the lacrimal sac: especially about its tumor cells [in Japanese]. Rinsho Ganka (Jpn J Clin Ophthalmol). 1956;10:477-482.

[26] Yamade S Kitagawa A. Malignant melanoma of the lacrimal sac. Ophthalmologica. 1978;177:30-33. 714368 10.1159/000308733

[27] Kuwana Y Suzuki H Taniuchi O, et al A case of malignant melanoblastoma occurred from the lacrimal sac [in Japanese]. Nippon Ganka Kiyo (Folia Ophthalmol Jpn). 1979;30:463-469.

[28] Uchida T Majima Y Shigemitsu T. A case of malignant melanoma of the lacrimal sac [in Japanese]. Ganka Rinsho Iho (Jpn Rev Clin Ophthalmol). 1990;84:2026-2029.

[29] Takahashi H Takigawa S Hamaguchi H Uji Y. A case of malignant melanoma of the lacrimal sac [in Japanese]. Ganka Rinsho Iho (Jpn Rev Clin Ophthalmol). 1991;85:470-472.

[30] Matsune S Uchizono A Shima T Kiyota R Furuta S Oyama M. Two cases of malignant melanoma originated primarily in the lacrimal sac and the mandibular bone [in Japanese]. Jibi-inkoka Tenbo (Otorhino Laryngol Tokyo). 1992;35:229-235.

[31] Matsuo H Inoue M Hanioka K Ito H. A case of primary malignant melanoma of the orbit [in Japanese]. Rinsho Ganka (Jpn J Clin Ophthalmol). 1993;47:406-407.

[32] Kuwabara H Takeda J. Malignant melanoma of the lacrimal sac with surrounding melanosis. Arch Pathol Lab Med. 1997;121:517-519. 9167609

[33] Goto R Hoshikawa H Mori N. Eight cases of malignant melanoma of the head and neck [in Japanese]. Jibi Inkoka Rinsho (Practica Otorhino Laryngol). 1999;92:1007-1011.

[34] Ito F Suga T Minakata T Ogawa Y. Treatment for 4 cases of lacrimal sac malignant tumors [in Japanese]. Nippon Keisei Geka Gakkai Kaishi (J Jpn Soc Plastic Reconstruct Surg). 2004;24:112-119.

[35] Shinozaki T Yamazaki M Hayashi R, et al Malignant melanoma of the lacrimal sac [in Japanese]. Tokeibu Geka (J Jpn Soc Head Neck Surg). 2005;15:203-206.

[36] Nakamura S Takeda K Tanaka R Araki Y. Two cases of malignant melanoma of the lacrimal sac [in Japanese]. Nippon Ganka Kiyo (Folia Ophthalmol Jpn). 2007;58:561-566.

[37] Maegawa J Yasumura K Iwai T Hata M Inayama Y Kobayashi S. Malignant melanoma of the lacrimal sac: a case report. Int J Dermatol. 2014;53:243-245. 24261959 10.1111/j.1365-4632.2012.05480.x

[38] Robert C Grob JJ Stroyakovskiy D, et al Five-year outcomes with dabrafenib plus trametinib in metastatic melanoma. N Engl J Med. 2019;381:626-636. doi:10.1056/NEJMoa1904059 31166680

[39] Rossi E Maiorano BA Pagliara MM, et al Dabrafenib and trametinib in BRAF mutant metastatic conjunctival melanoma. Front Oncol. 2019;9:232. 31024839 10.3389/fonc.2019.00232 PMC6460374

[40] Matsuo T Ogino Y Ichimura K Tanaka T Kaji M. Clinicopathological correlation for the role of fluorodeoxyglucose positron emission tomography computed tomography in detection of choroidal malignant melanoma. Int J Clin Oncol. 2014;19:230-239. 23456141 10.1007/s10147-013-0538-5

